# A simplified low-cost and reliable plant genomic DNA extraction method for PCR-based genotyping and screening

**DOI:** 10.1186/s13007-025-01480-8

**Published:** 2025-12-10

**Authors:** Cai-yun Yang, Duncan Scholefield, Stephen Ashling, Surbhi Grewal, Ian P. King, Julie King

**Affiliations:** Nottingham Wheat Research Centre, School of Biosciences, Sutton Bonington Campus, University of Nottingham, Loughborough, LE12 5RD UK

**Keywords:** Plant genomic DNA extraction, KASP (Kompetitive Allele-Specific PCR), PCR (polymerase chain reaction)

## Abstract

**Background:**

Extraction of plant genomic DNA is a critical step for PCR-based genotyping, mapping, and breeding applications. Conventional CTAB protocols and commercial kits provide reliable DNA but are labour-intensive, costly, and generate substantial plastic waste. Simplified crude-extract methods are available, yet their performance is often compromised by PCR inhibition from salts and cellular debris. A rapid, low-cost, and high-throughput method is therefore needed for routine molecular applications.

**Results:**

We developed a single-tube DNA extraction protocol that eliminates supernatant transfers, thereby reducing handling errors, plastic consumption, and processing time. The method consistently produces DNA of sufficient yield and purity for PCR-based assays. Validation in wheat and wheat–wild relative introgression lines demonstrated robust amplification in KASP assays. Cross-species testing in maize, Arabidopsis, and tomato using two Tris-salt extraction buffers confirmed broad applicability, supported by NanoDrop and Qubit measurements. Freeze-dried and frozen tissue produced higher yields than fresh samples, confirming their suitability for high-throughput and large-scale studies.

**Conclusions:**

This streamlined protocol provides a cost-effective, reliable, and scalable approach for extracting plant genomic DNA suitable for PCR-based genotyping, marker development, and diversity analysis. Its simplicity and throughput make it particularly valuable for breeding programmes, although it is not intended for applications requiring highly pure DNA, such as whole-genome resequencing.

**Supplementary Information:**

The online version contains supplementary material available at 10.1186/s13007-025-01480-8.

## Background

Extraction of plant genomic DNA is an essential step for downstream molecular applications in genetics and breeding. Polymerase chain reaction (PCR)-based analyses such as genotyping, mapping, and screening require reliable, scalable, and cost-effective DNA isolation methods that can handle large sample numbers. Commercial extraction kits yield high-quality DNA but are expensive at scale. The cetyltrimethylammonium bromide (CTAB) extraction protocol of Doyle and Doyle [6] and its many modifications remain widely used for plant DNA extraction [1, 2, 4, 5, 7, 9, 21]. These methods, however, typically involve multiple transfers of DNA-containing supernatants into fresh tubes, which increases handling errors, time, and the consumption of plastic consumables, particularly when processing large sample numbers [23].

Several simplified non-commercial protocols have also been reported for plant DNA extraction [16, 17, 20, 28]. Many rely on crude extracts or neutralised lysates that are pipetted directly into PCR reactions [12, 26, 27], and in some cases, even small amounts of plant tissue have been added directly to the PCR mix [3]. While these approaches reduce processing steps, their effectiveness is often constrained by the tolerance of PCR assays to inhibitory substances such as salts, pH fluctuations, or cellular debris present in the extracts.

To overcome these limitations, we established a reliable, high-throughput DNA extraction protocol that combines speed, low cost, and robustness. The method eliminates the need for supernatant transfer, enabling all steps to be performed within a single tube or a 96-well plate format. This design streamlines the workflow, reduces handling errors and plastic consumption, and allows up to 96 samples to be processed in parallel within a few hours. The protocol consistently produces DNA of sufficient yield and purity for PCR-based genotyping and mapping applications. Its reliability was demonstrated across diverse plant species including wheat, maize, Arabidopsis, and tomato, highlighting its broad applicability. Importantly, the procedure does not include RNase A treatment or phenol-based purification and is specifically optimised for PCR-based genotyping rather than sequencing-based applications such as whole-genome resequencing.

## Materials and methods

**Fig. 1:**
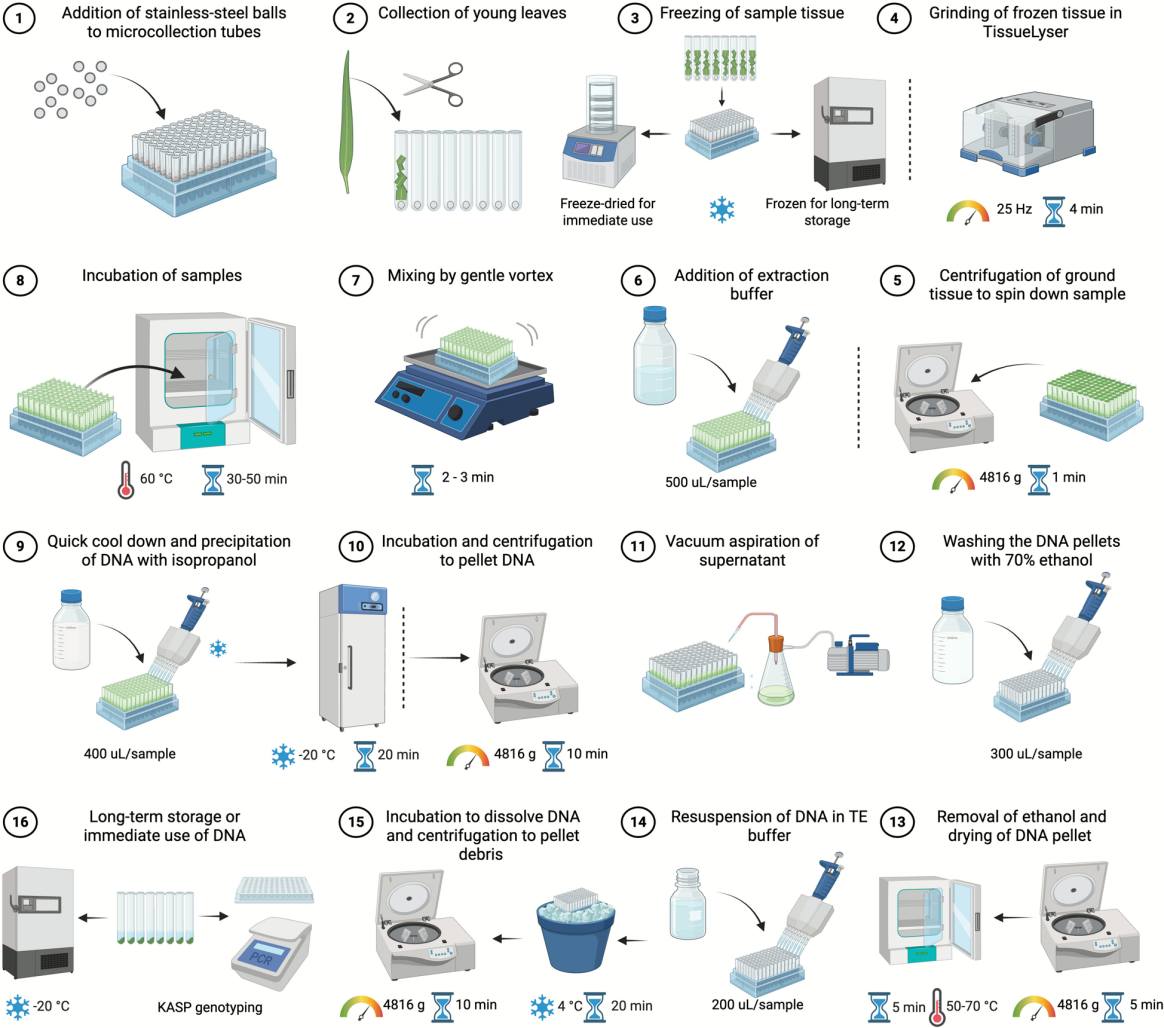
A graphical summary of the DNA extraction method

A graphical summary of the full DNA extraction method is shown in Fig. 1.

### Plant material

Young leaves (20 to 70 mg with 50 mg as optimum, corresponding to six segments of ~ 1 cm each) were collected into 8-tube strip collection microtubes and capped with collection microtube 8-cap strips (QIAGEN, Cat. nos. 19560 and 19,566), each pre-loaded with a 4.5 mm stainless-steel ball bearing (Amazon). The tubes were arranged in a 12 × 8 rack format to enable simultaneous processing of up to 96 samples. Tissue was stored at− 20 °C for short-term use, at− 80 °C for long-term storage, or freeze-dried overnight prior to extraction [the same amount of leaf material (20 to 70 mg) was used for both frozen and freeze-dried]. For cross-species validation, tissue was collected from wheat, maize, Arabidopsis and tomato under the same conditions.

### Reagents and buffers

Two simple salt-based extraction buffers were evaluated. The recommended buffer was TPS (100 mM Tris–HCl, pH 9.5; 1 M KCl; 10 mM EDTA; [27]). As a practical alternative, a NaCl buffer (100 mM Tris–HCl, pH 8.0,1.5 M NaCl; 20 mM EDTA), adapted from Doyle and Doyle [6], was also tested and found to perform equivalently. Additional reagents included isopropanol, 70% (v/v) ethanol, TE buffer (10 mM Tris–HCl, pH 8.0,1 mM EDTA), and DNase-free water.

### Equipment

Tissue was disrupted using a TissueLyser II (QIAGEN). Centrifugation was performed in a bench-top centrifuge with a rotor designed to accommodate 96-well plate footprints, allowing the racked 8-tube strips to be processed directly. The maximum centrifugal force achievable with this rotor was 4816 × *g* (approximately 4700 rpm), which defined the conditions used throughout. For laboratories using single tubes rather than the 96-tube system, extractions should be performed at the maximum RCF supported by the rotor. For single-tube work, 2 mL round-bottom microcentrifuge tubes (Sarstedt Ltd, Cat. no. 72.695.400) are recommended, as the 4.5 mm bead does not reach the bottom of 1.5 mL tubes. Removal of the supernatant from the nucleic acids (which were pelleted with cellular debris) during the extraction was carried out using a vacuum aspirator (Figure S1), which is strongly recommended for high-throughput and error-free handling. Where a vacuum system is not available, the supernatant can be removed by pipette.

### DNA extraction

Frozen or freeze-dried tissue was ground at 25 Hz for 4 min using the TissueLyser II. Tubes were briefly centrifuged at 4816 × g for 1 min to collect powder from the caps. Extraction buffer (500 µL) was added, and samples were mixed by gentle inversion or vortexing before incubation at 60 °C for 30–50 min (30 min minimum but upto 50 min for convenience). Following a 5 min cool-down, isopropanol (0.8 volumes of Extraction buffer; 400 µL) was added, mixed, and incubated for a minimum of 20 min. For 8-tube strip microtubes with 8-cap strips, incubation was carried out at − 20 °C to prevent ballooning of the caps after the preceding heat step in combination with isopropanol. For 2 mL round-bottom microcentrifuge tubes with hinged attached caps, incubation at room temperature was sufficient and gave equivalent results. Samples were centrifuged at 4816 × *g* for 10 min to pellet nucleic acids together with plant debris. The supernatant was carefully removed using either a pipette or a vacuum aspirator.

Pellets were washed with 300 µL of 70% ethanol, gently vortexed, and centrifuged again at 4816 × g for 5 min. Ethanol was removed, and the pellets were dried at 70 °C for 5 min to evaporate any remaining ethanol. This temperature can be lowered and the time for evaporation increased to reduce any heat denaturation of the DNA, although the downstream application was not affected by the 70 °C. Dried pellets were resuspended in 200 µL of TE buffer and incubated on ice or at 4 °C for 20 min, followed by gentle vortexing. Samples were centrifuged at 4816 × *g* for 10 min to pellet debris, leaving DNA in the supernatant. The recovered DNA was used directly for PCR and KASP assays or stored at − 20 °C. For high-throughput genotyping, a 1:20 dilution of the extract was routinely used. A full 96-well plate could be processed in approximately 3–4 h, from grinding to DNA suitable for PCR.

Although TE buffer was used as the standard resuspension solution to provide long-term stability, nuclease-free water can be used where DNA is intended for short-term storage or immediate PCR use, as reported in other published protocols. Care should be taken when TE is used as template for PCR, as EDTA can inhibit amplification through chelation of Mg^2^⁺ ions [24].

### Validation and comparisons

DNA was extracted from 50 mg fresh, frozen and freeze-dried wheat leaf tissue and DNA stored at − 20 °C for four years to compare quantity and purity across tissue types. TPS and NaCl buffers were tested in wheat, maize, Arabidopsis and tomato. For these cross-species tests, extractions were performed in 2 mL round-bottom tubes as described above. DNA concentration and purity were measured using a NanoDrop 2000 spectrophotometer (Thermo Fisher Scientific) and Qubit 2.0 Fluorometer with the Qubit dsDNA BR Assay Kit (Thermo Fisher Scientific, Cat. no. Q32853). DNA integrity was assessed by electrophoresis of 3 µL of extract on 1% (w/v) agarose gels prepared in 1 × TAE buffer (40 mM Tris–acetate, 1 mM EDTA, pH 8.0) containing ethidium bromide (0.5 µg/mL). HyperLadder 1 kb (Cat. no. BIO33025, Scientific Laboratory Supplies) was used as the molecular weight marker. Gels were visualised under UV light.

### PCR and KASP validation

PCR amplification was carried out using species-specific primers (Table 1). Each 20 µL reaction contained 5 µL of DNA extract (diluted 1:20 in water). Cycling conditions were as follows: 96 °C for 5 min; 35 cycles of 94 °C for 20 s, 56 °C for 20 s, and 72 °C for 40 s; followed by a final extension at 72 °C for 2 min. PCR products (5 µL from each reaction) were visualised on 1% agarose gels. DNA extractions from wheat and wheat-wild relative introgression lines were also validated for use in Kompetitive Allele-Specific PCR (KASP) assays following the genotyping protocols described by Grewal et al. [15].

**Table 1:** Species-specific primers used to check the extracted DNA

Species	Species specific primers
Wheat	5’-TGG CAC CCT CAA TGT AGA C-3’ 5’-GCT TGC CCA TTT CAC AAC-3’
Maize	5’-TCAGAGCCAGTACAAGAGAGT-3’ 5’-TGGAGCGATCACCATAACGT-3’
Arabidopsis	5’-GCAATCTCTCATTCCGATAGTC-3’ 5’-CGAAATACCGAACATCAACATC-3’
Tomato	5’-TCGTTCGTCTGACTTGGGTAT-3’ 5’-GATTCGGCAGGTGAGTTGTTA-3’

### Statistical analysis

DNA concentration and purity were reported as descriptive statistics (mean and range) based on replicate extractions. No formal hypothesis testing was performed, as the aim was method validation rather than statistical inference.

## Results

### Suitability of DNA extracts for KASP genotyping

**Fig. 2:**
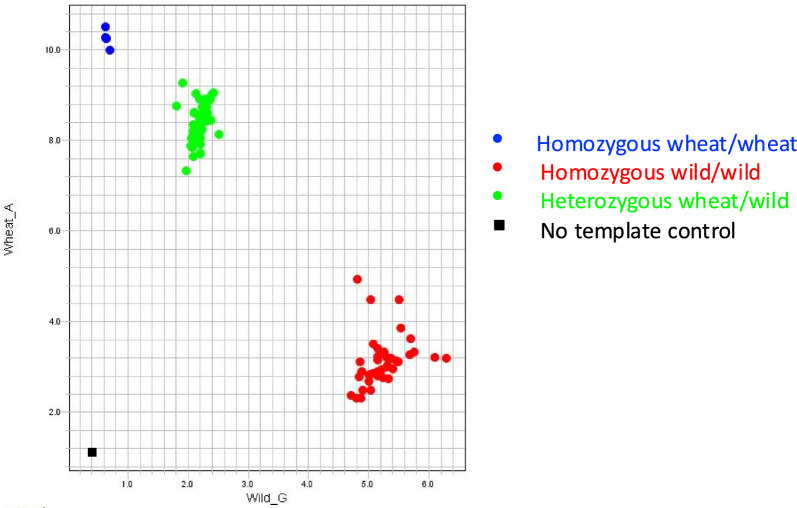
KASP genotyping of wheat-wild relative introgression lines

Initial tests with crude salt-based extraction approaches [27, 28] produced inconsistent amplification in KASP assays, indicating that DNA purity was insufficient for reliable use in this application. To address this, we incorporated an isopropanol precipitation step to remove residual buffer components, followed by resuspension of DNA in either TE buffer or nuclease-free water. This modification enabled robust and reproducible KASP amplification. The resulting DNA extracts supported successful marker development and high-throughput genotyping of wheat–wild relative introgression lines [15], Fig. 2). Clear separation of allele clusters in the KASP plot (Fig. 2) validated the suitability of the extracted DNA for downstream genotyping. For routine use, a 1:20 dilution of the DNA extract was effective, with 5 µL of diluted supernatant per PCR reaction sufficient to generate approximately 600–700 reactions from ~ 200 µL of extract derived from ~ 50 mg of leaf tissue.

Allelic discrimination plot generated using QuantStudio™ 5 (Thermo Fisher Scientific) for KASP marker WRC2039 [15], used to screen wheat-wild relative introgression lines. Each point represents an individual sample, with fluorescence intensity plotted for the wheat-specific allele (y-axis) against the wild relative-specific allele (x-axis). Blue clusters represent homozygous wheat genotypes, red clusters indicate homozygous wild genotypes, and green clusters correspond to heterozygous plants carrying both wheat and wild alleles. Black squares denote no-template controls (NTCs).

### DNA yield from fresh, frozen and freeze-dried tissue

DNA was extracted from wheat leaves under four conditions: fresh, frozen, stored at -20 °C for four years and freeze-dried. NanoDrop concentrations ranged from 117 to 203 ng/µL, with 260/280 ratios between 1.76 and 2.05, consistent with high-quality DNA (Table 2). Qubit measurements were lower (17.3–87.8 ng/µL), reflecting the more specific detection of double-stranded DNA. Among the tissue types, frozen and freeze-dried samples yielded higher concentrations than fresh tissue.

**Table 2:** DNA concentrations of DNA extracted from fresh, frozen, stored at -20 °C for 4 years and freeze-dried young wheat leaves

Sample No	Sample tissue	Nanodrop (ng/μl)	A260/280	A260/230	Qubit (ng/μl)	Total vol. (μl)	Total amount DNA (µg)
1	Fresh leaves	140.3	1.76	0.89	17.3	200	34.6
2	Fresh leaves with extraction buffer	117	1.85	1.08	28.6	200	57.2
3	Frozen (-70 °C) leaves	203	1.88	1.51	59.7	200	119.4
4	Freeze dried leaves	131.8	1.83	1.31	47.8	200	95.6
5	DNA stored at -20 °C for 4 years	442.2	2.05	1.85	87.8	200	175.6

### Cross-species validation of the DNA extraction protocol

The DNA extraction method was evaluated on wheat (*Triticum aestivum*), maize (*Zea mays*), *Arabidopsis thaliana* and tomato (*Solanum lycopersicum*) using TPS and NaCl buffers. NanoDrop concentrations ranged from 160.7 ng/μl (*Arabidopsis*, NaCl) to 1118.2 ng/μl (tomato, TPS), with A260/280 ratios close to the expected range of 1.8–2.0 and A260/230 ratios generally > 1.1 (Table 3). Qubit readings were consistently lower, reflecting the exclusion of RNA and other contaminants, but confirmed that yields were sufficient for downstream applications.

**Table 3:** DNA yield and quality from 50 mg freeze-dried leaf tissue from wheat, maize, Arabidopsis and tomato. Leaf material was extracted using two buffers into 2 mL microcentrifuge tubes

Sample number	Plant species	Extraction buffer	Nanodrop ng/μl	A260/280	A260/230	Qubit ng/μl	Total vol. (μl)	Total DNA (µg)
1	Wheat	TPS	427.1	2.03	1.71	83.8	200	167.6
2	Wheat	NaCl	310.9	1.97	1.39	86.8	200	173.6
3	Maize	TPS	571	2.06	1.52	59.8	200	119.6
4	Maize	NaCl	490.4	2.05	1.64	53.2	200	106.4
5	Arabidopsis	TPS	215	1.90	1.14	7.1	200	14.2
6	Arabidopsis	NaCl	160.7	2.11	1.27	8.3	200	16.6
7	Tomato	TPS	1118.2	1.97	1.46	100	200	200
8	Tomato	NaCl	793.9	1.91	1.50	98	200	196

Concentrations were measured using a Nanodrop and a Qubit

**Fig. 3:**
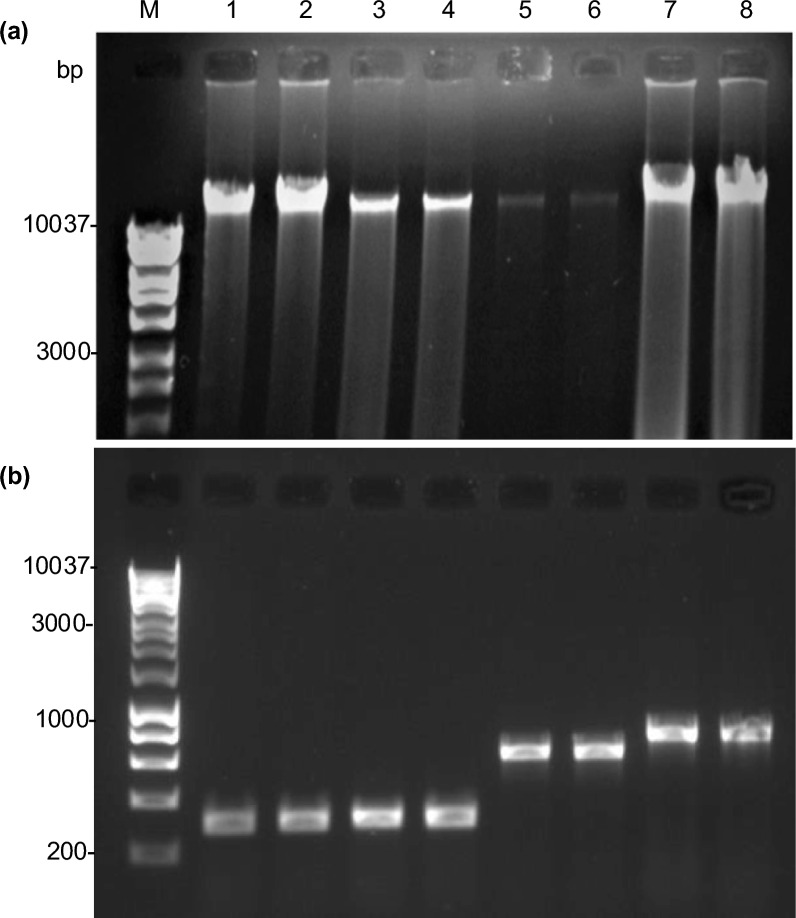
Validation of DNA quality across four species using TPS and NaCl buffers. **a** Visualization of extracted DNA on 1% agarose gel (3 μL of extract per lane) **b** PCR amplification of species-specific primers using extracted DNA visualized on a 1% agarose gel (5 μL of PCR product per lane). Lanes: M, 1 kb DNA ladder; 1 and 2, Wheat; 3 and 4, Maize; 5 and 6, Arabidopsis; 7 and 8, Tomato. Odd-numbered lanes: TPS buffer; even-numbered lanes: NaCl buffer

DNA extracted using both buffers was intact and clearly visible on agarose gels (Fig. 3a). Despite variation in yields across species (Table 3), PCR using a 1:20 dilution of the DNA extracts produced strong amplification with species-specific primers (Table 1) in all four species (Fig. 3b). This demonstrates that the extracted DNA, even when diluted, retained adequate integrity and purity for PCR-based genotyping. Together with the quantitative data (Table 3), these results confirm that even simple salt-based extraction buffers can produce DNA that is both stable and functional across diverse species.

### Low cost

DNA extracted using this method was calculated to cost £0.99 per sample when using 12 × 8-tube strip collection microtubes and £0.11 when using 2 mL microcentrifuge tubes. This compares with a per sample cost of commercial DNA extraction kits such as Qiagen (DNeasy Plant Mini kit) of £3.68 and Sigma (Gen Elute Plant Genomic DNA Miniprep kit) of £3.69. (Table S1 gives the breakdown of the cost calculation). The cost of the commercial kits quoted does not include extra consumables such as pipette tips.

## Discussion

The DNA extraction protocol described here builds upon simplified salt-based methods while addressing their limitations for high-throughput applications. By incorporating an isopropanol precipitation and resuspension step, the protocol removes residual polysaccharides, salts, and proteins, substantially improving DNA quality for PCR-based assays. This modification enables consistent amplification in KASP genotyping, overcoming the variability observed when crude extracts are used directly (Thomson and Henry, 1995). The streamlined workflow ensures that the method remains rapid and scalable, supporting reliable processing of large sample sets, as demonstrated in wheat–wild relative introgression studies [10, 11, 13–15, 18, 19].

Tissue pre-treatments were found to strongly influence recovery. Frozen and freeze-dried leaves yielded higher DNA concentrations than fresh material, reflecting more efficient cell disruption through ice-crystal formation during freezing or micro-porosity generated during drying [22, 29]. Freeze-drying in particular offers the dual advantages of stable long-term storage and improved DNA release, making it especially valuable for high-throughput genotyping pipelines and collaborative projects where samples may need to be processed at different times or sites. Fresh leaf tissue was included only for validation, and the optimised protocol is explicitly designed to begin with frozen or freeze-dried material as the preferred starting point. DNA stored at − 20 °C for four years was checked for quantity and quality on an agarose gel and also used in a PCR amplification with wheat specific primers. The quality and amplification were very similar to the newly extracted samples (Table 2), showing the DNA extracted using the method described here is suitable for at least medium-term storage (in the lab, plates stored for 7 to 8 years have been used for KASP genotyping when new primers have been designed, with successful results).

Spectrophotometric (NanoDrop) and fluorometric (Qubit) measurements provided complementary views of DNA output, with NanoDrop typically reading higher because it captures RNA and other solutes, whereas Qubit quantifies double-stranded DNA. While both these measurements were used in this study to compare DNA yield and quality across treatments, such quantification is not essential for routine use of the protocol. In practice, extracts can be used directly for PCR without prior quantification. If quality control is required, a quick NanoDrop measurement or random sampling of extracts provides sufficient information for most genotyping workflows.

Cross-species validation confirmed the robustness and versatility of the method. Despite variation in yield among wheat, maize, Arabidopsis, and tomato, DNA extracted using both TPS and NaCl buffers was intact, PCR-amplifiable, and suitable for genotyping. This demonstrates that complex additives such as CTAB and polyvinylpyrrolidone (PVP), which are frequently included in traditional protocols [6, 21], are not required for reliable PCR-based applications. The simplified salt-based formulation, coupled with the precipitation step, is sufficient to deliver consistent and reproducible results across diverse species.

Together, these results highlight that the optimised method provides a practical and cost-effective alternative to commercial kits (at less than one third the cost per sample) and more elaborate extraction protocols. By reducing consumable use, minimising handling, and maintaining throughput while ensuring reliability, this approach directly addresses the needs of large-scale molecular breeding programmes and population studies. For applications requiring long-read sequencing or chemically intact DNA, additional steps such as RNase treatment, phenol-free clean-up, or high molecular weight extraction strategies may be necessary, and should be evaluated systematically.

## Conclusions

This streamlined DNA extraction method is simple, reliable, and high-throughput, providing sufficient yield and purity for PCR and KASP genotyping. The entire procedure is performed in a single tube, minimising labelling errors, plastic waste, and sample handling. The inclusion of an isopropanol precipitation step greatly enhances DNA quality, while pre-treatments such as freeze-drying ensure high recovery without compromising integrity.

The method’s compatibility with diverse plant species and its successful application in large-scale wheat–wild relative introgression studies highlight its practical value for breeding, mapping, and genetic diversity analyses. By reducing reagent complexity and handling steps while maintaining throughput, this protocol offers an effective alternative to more elaborate extraction methods and provides an accessible tool for high-throughput genotyping in plant research and breeding programmes.

## Supplementary Information

Supplementary material 1. Figure S1. Vacuum aspirator setup. A simple vacuum aspirator system used to remove supernatant and waste solutions during DNA extraction. This setup allows rapid processing of large sample numbers in 96-well plates, minimises pipette tip changes, and reduces plastic waste.

Supplementary material 2. Table S1. Cost comparison of this DNA extraction method and commercial kits.

## Data Availability

Data sharing is not applicable to this article as no datasets were generated or analysed during the current study.
