# Supplementary figures and images for: A simplified low-cost and reliable plant genomic DNA extraction method for PCR-based genotyping and screening
Source: Plant Methods. 2025 Dec 10;22:2. doi: 10.1186/s13007-025-01480-8 (PMC12801886; doi:10.1186/s13007-025-01480-8)

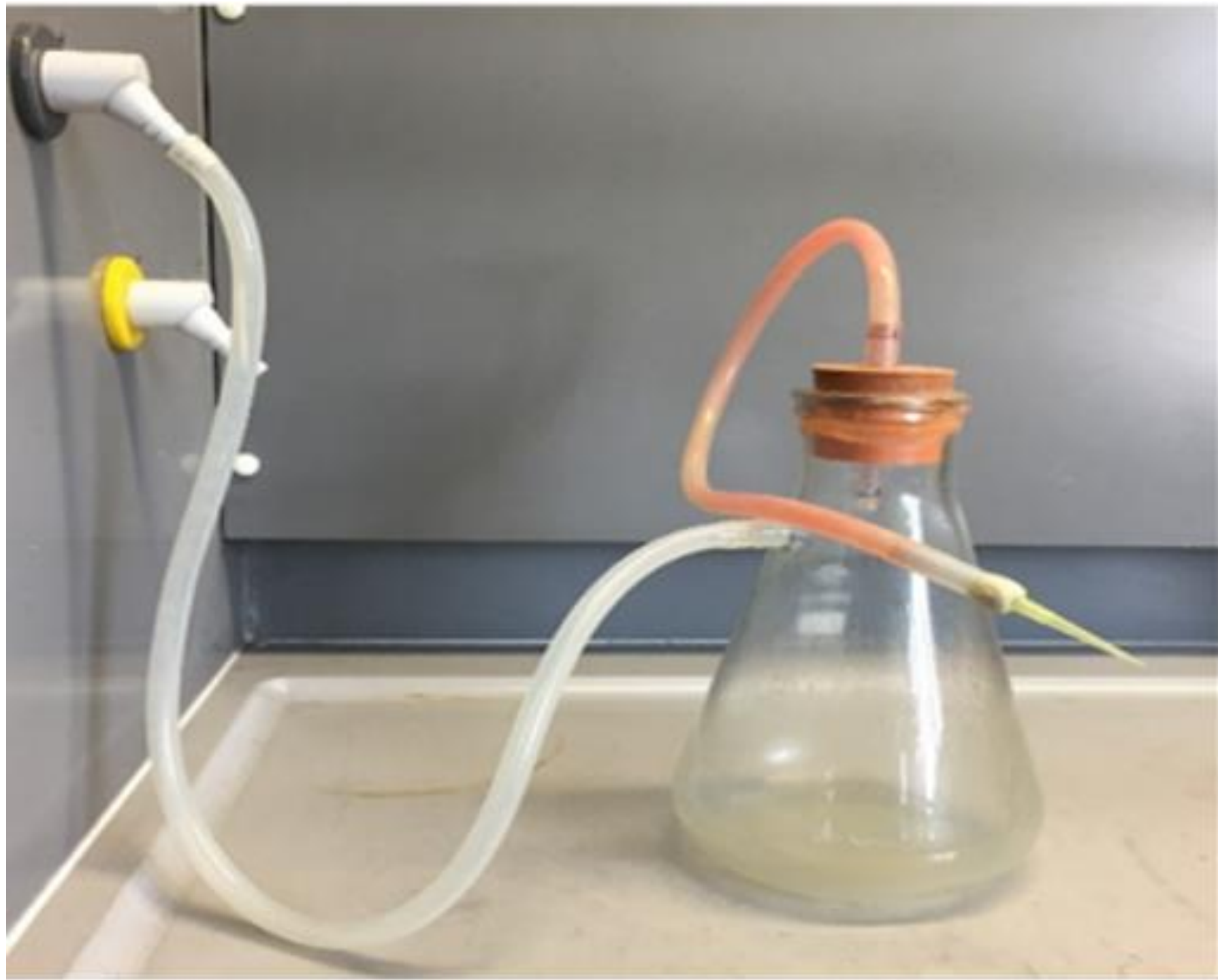

Supplement: Supplementary file 1 — Supplementary material 1. Figure S1. Vacuum aspirator setup. A simple vacuum aspirator system used to remove supernatant and waste solutions during DNA extraction. This setup allows rapid processing of large sample numbers in 96-well plates, minimises pipette tip changes, and reduces plastic waste. [file 13007_2025_1480_MOESM1_ESM.pdf]
